# Supplementary material for: Quantitative Signatures of Disassembly Mechanisms Modulating Filament and Bundle Assembly in a Shared Pool
Source: bioRxiv. 2025 May 28:2025.05.24.655867. Preprint. [Version 1] doi: 10.1101/2025.05.24.655867 (PMC12154648; doi:10.1101/2025.05.24.655867)
Supplement: Supplementary file 1 [file NIHPP2025.05.24.655867v1-supplement-1.pdf]

bioRxiv preprint doi: https://doi.org/10.1101/2025.05.24.655867; this version posted May 28, 2025. The copyright holder for this preprint (which was not certified by peer review) is the author/funder, who has granted bioRxiv a license to display the preprint in perpetuity. It is made available under aCC-BY 4.0 International license.

Supplementary Material

Derivation of Mean Length Dynamics for Bare Filament and Bundled Structures

Bare Filament

Assembly of a bare filament with severing in a limited pool: Solving Equation 2 in the main text, with a growth rate $k_+^{'}(N-L)$, a severing rate s, and initial conditions $t=0$, $L=0$, the time evolution of filament length is given by $L(t) = L_{\text{ss}}\tanh(k_{\text{LPS}}t)$, where the steady-state length and relaxation rate are given by $L_{\text{SS}} = \frac{\pi k_+^{'}}{4s}\left(\sqrt{1+\frac{8sN}{\pi k_+^{'}}}-1\right)$ and $k_{\text{LPS}} = \frac{k_+^{'}}{2}\cdot\sqrt{\pi\left(1+\frac{8sN}{\pi k_+^{'}}\right)}$, respectively. This equation is plotted in Figure S1B.

For large values of N, where $\frac{8sN}{\pi k_+^{'}} \gg 1$, the relaxation rate simplifies to $k_{\text{LPS}} \approx k_{\text{S}}$, which is equivalent to the rate k_{S} found in the severing scenario with a free monomer pool.

Bundled Structure

Assembly of a bundle with severing in a free pool: Here, the length dynamics of an individual filament in a bundle of n filaments are described by

$$\frac{\mathrm{d}L_i}{\mathrm{d}t} = \frac{k_+'}{n}N - \frac{2}{\pi n}sL_i^2, \quad i = 1, \ldots, n, \tag{3}$$

where $\frac{k_+'}{n}N$ and $\frac{s}{n}$ represent the Identical growth and severing rates for individual filaments, while $k_+^{'}N$ and s represent the growth and severing rates for the sum total of n filaments. Assuming equal filament lengths $L_i = L_{\text{avg}}$, the average length evolves as

$$\frac{\mathrm{d}L_{\text{avg}}}{\mathrm{d}t} = \frac{k_+^{'}}{n}N - \frac{2}{\pi n}sL_{\text{avg}}^2. \tag{4}$$

With initial conditions at $t=0$, $L_{\text{avg}}=0$, the time evolution of filament length is given by $L_{\text{avg}}(t) = L_{\text{SS}}\tanh(k_{\text{S}}t)$, where the steady-state length and relaxation rate are $L_{\text{SS}} = \sqrt{\frac{\pi k_+^{'}N}{2s}}$ and $k_{\text{S}} = \frac{1}{n}\sqrt{\frac{2k_+^{'}Ns}{\pi}}$, respectively. This equation is used to calculate the autocorrelation function of average filament length at steady state.

Assembly of a bundle with severing in a limited pool: Here, the length dynamics of an individual filament in a bundle of n filaments are described by

$$\frac{\mathrm{d}L_i}{\mathrm{d}t} = \frac{k_+'}{n}\left(N - \sum_{f=1}^{n} L_f\right) - \frac{2}{\pi n}sL_i^2, \quad i = 1, \ldots, n, \tag{5}$$

where, $\frac{k_+'}{n}(N-L)$ and $\frac{s}{n}$ denote the identical growth and severing rates of the individual filaments, whereas $k_+^{'}(N-L)$ and s correspond to the growth and severing rates for the sum total

bioRxiv preprint doi: https://doi.org/10.1101/2025.05.24.655867; this version posted May 28, 2025. The copyright holder for this preprint (which was not certified by peer review) is the author/funder, who has granted bioRxiv a license to display the preprint in perpetuity. It is made available under aCC-BY 4.0 International license.

of n filaments. Assuming equal filament lengths $L_i = L_{\text{avg}}$, the average length evolves as

$$\frac{\mathrm{d}L_{\text{avg}}}{\mathrm{d}t} = \frac{k'_+}{n}(N - nL_{\text{avg}}) - \frac{2}{\pi n} s L_{\text{avg}}^2. \tag{6}$$

Starting with the initial condition $L_{\text{avg}}(0) = 0$, the filament length evolves over time according to $L_{\text{avg}}(t) = L_{\text{SS}} \tanh(k_{\text{LPS}} t)$, where the steady-state length and the relaxation rate are given by $L_{\text{SS}} = \frac{\pi k'_+ n}{4s}\left(\sqrt{1 + \frac{8sN}{\pi k'_+ n^2}} - 1\right)$ and $k_{\text{LPS}} = \frac{k'_+}{2n}\sqrt{\pi\left(1 + \frac{8sN}{\pi k'_+ n^2}\right)}$, respectively. This expression is used to calculate the autocorrelation function of the average filament length at steady-state.

For large N such that $\frac{8sN}{\pi k'_+} \gg 1$, the relaxation rate k_{LPS} approximates k_{S}, matching the relaxation rate observed in the severing scenario with a free monomer pool.

Probability Distribution of Lengths of Bare Filaments and Bundled Structures

The detailed analytical derivations for the probability distributions of bare filaments and the coassembly of multiple filaments under the constant disassembly mechanism within a limited pool are provided in our previous work [32]. The derivations corresponding to severing dynamics in a free pool are presented in our earlier studies [61, 64]. Furthermore, the analytical derivations for the probability distributions of bundles undergoing constant disassembly and severing in a free pool is discussed in [46]. In the present work, we extend these analyses by deriving the length probability distributions for both bare filaments and bundles regulated by severing within a limited pool.

Probability Distribution of Lengths of Bare Filaments Under Constant Disassembly in a Limited Pool

The master equation governing the probability $P(L,t)$ for a filament of length L under a limited pool with severing is:

$$\frac{dP(L,t)}{dt} = k'_+(N-L+1)P(L-1,t) + s\sum_{i=L+1}^{\infty} P(i,t) - k'_+(N-L)P(L,t) - s(L-1)P(L,t) \tag{7}$$

At steady state, where $\frac{dP(L,t)}{dt} = 0$, and applying the normalization condition $\sum_{i=1}^{\infty} P(i) = 1$, we define $\rho = \frac{k'_+}{s}$. Using the recursive method from our previous work [61], the probability distribution for $L \geq 0$ is given by

$$P(L) = \frac{L\rho^{L-1}}{N-L}\prod_{i=1}^{L}\frac{N-i}{\rho(N-i)+i}. \tag{8}$$

This equation is plotted in Figure S1D. To simplify, we rewrite the product and approximate it for large $\rho(N-i)$. For $L \ll N$, this yields the approximate probability distribution $P(L) \approx$

bioRxiv preprint doi: https://doi.org/10.1101/2025.05.24.655867; this version posted May 28, 2025. The copyright holder for this preprint (which was not certified by peer review) is the author/funder, who has granted bioRxiv a license to display the preprint in perpetuity. It is made available under aCC-BY 4.0 International license.

$\frac{L}{\rho N} \exp\left(-\frac{L^2}{2\rho N}\right)$. Substituting $\rho = \frac{k'_+}{s}$, we obtain the final form:

$$P(L) = \frac{sL}{k'_+ N} \exp\left(-\frac{sL^2}{2k'_+ N}\right). \tag{9}$$

Probability Distribution of Individual Filament Lengths in a Bundle of n Filaments Regulated by Severing in a Limited Pool

First, we consider two filaments, L_1 and L_2, growing in the pool. The master equations for these two filaments are given by:

$$\begin{aligned}\frac{dP(L_1, L_2, t)}{dt} &= k'_+ \left((N - L_2) - L_1 + 1\right) P(L_1 - 1, L_2, t) + s \sum_{i=L_1+1}^{\infty} P(i, L_2, t)\\ &- k'_+ \left((N - L_2) - L_1\right) P(L_1, L_2, t) - s(L_1 - 1)P(L_1, L_2, t)\\ &+ k'_+ \left((N - L_1) - L_2 + 1\right) P(L_1, L_2 - 1, t) + s \sum_{i=L_2+1}^{\infty} P(L_1, i, t)\\ &- k'_+ \left((N - L_1) - L_2\right) P(L_1, L_2, t) - s(L_2 - 1)P(L_1, L_2, t).\end{aligned} \tag{10}$$

When the total number of monomers N is very large, the influence of one filament's average length on the average length of other filaments becomes negligible. As a result, at any given time, the length of one filament (say L_1) depends primarily on the available number of free monomers, which is given by $N^* - L_1 = N - \langle L_2 \rangle - L_1$, where $N^* = N - \langle L_2 \rangle$ and $\langle L_2 \rangle$ is the steady-state average length of L_2. Under these conditions, the master equation for individual filament L_1 simplifies to:

$$\frac{dP(L_1, t)}{dt} = k'_+(N^* - L_1 + 1)P(L_1 - 1, t) + s \sum_{i=L_1+1}^{\infty} P(i, t) - k'_+(N^* - L_1)P(L_1, t) - s(L_1 - 1)P(L_1, t) \tag{11}$$

At steady state, i.e., when $\frac{dP(L,t)}{dt} = 0$, and using the normalization condition $\sum_{i=1}^{\infty} P(i) = 1$, we define $\rho = \frac{k'_+}{s}$. Following the recursive approach outlined in our previous work [61], The resulting probability distribution for L_1 is then:

$$P(L_1) = \frac{L_1 \rho^{L_1 - 1}}{[N - \langle L_2 \rangle] - L_1} \prod_{i=1}^{L_1} \frac{N - \langle L_2 \rangle - i}{\rho(N - \langle L_2 \rangle - i) + i}, \tag{12}$$

This equation can be further simplified in a manner similar to Equation 9 as follows:

$$P(L_1) = \frac{sL_1}{k'_+[N - \langle L_2 \rangle]} \exp\left(-\frac{sL_1^2}{2k'_+[N - \langle L_2 \rangle]}\right), \tag{13}$$

To find the average filament length $\langle L_1 \rangle$, we use the expectation formula $\langle L_1 \rangle = \int_0^\infty L_1 P(L_1) dL_1$. Substituting $P(L_1)$ and simplifying, the integral reduces to a Gaussian form. Using the known integral $\int_0^\infty x^2 e^{-x^2} dx = \frac{\sqrt{\pi}}{4}$, we get $\langle L_1 \rangle = \frac{\sqrt{\pi}\sqrt{k'_+[N - \langle L_2 \rangle]}}{2\sqrt{s}}$. Assuming $\langle L_1 \rangle = \langle L_2 \rangle = \langle L \rangle$, the steady-state length satisfies $\langle L \rangle = \frac{\sqrt{\pi}\sqrt{k'_+[N - \langle L \rangle]}}{2\sqrt{s}}$. Solving this quadratic equation for the

bioRxiv preprint doi: https://doi.org/10.1101/2025.05.24.655867; this version posted May 28, 2025. The copyright holder for this preprint (which was not certified by peer review) is the author/funder, who has granted bioRxiv a license to display the preprint in perpetuity. It is made available under aCC-BY 4.0 International license.

positive root, we obtain

$$\langle L \rangle = \frac{-\pi k'_+ + \sqrt{(\pi k'_+)^2 + 16 s \pi k'_+ N}}{8s}. \tag{14}$$

Similarly, when there are n filaments $L_1, L_2, \ldots, L_n$ present in the pool and regulated by severing in a limited pool mechanism, we can generalize Equation 12 as:

$$P(L_1) = \frac{L_1 \rho^{L_1 - 1}}{[N - (n-1)\langle L_n \rangle] - L_1} \prod_{i=1}^{L_1} \frac{N - (n-1)\langle L_n \rangle - i}{\rho(N - (n-1)\langle L_n \rangle - i) + i} \tag{15}$$

where,

$$\langle L_n \rangle = \frac{-\pi k'_+ + \sqrt{(\pi k'_+)^2 + 16 s \pi k'_+ N}}{8s}. \tag{16}$$

This equation is plotted in Figure S5A.

Probability Distribution of the Bundle Length Consisting of n Filaments Regulated by Severing in a Limited Pool

For a bundle consisting of n filaments, each of length l_i, the filament lengths follow the distribution

$$p(l_i) = \frac{s l_i}{2k'_+ N} \exp\left(-\frac{s l_i^2}{2k'_+ N}\right). \tag{17}$$

The cumulative probability that a filament length is less than L is $F(L) = \int_0^L p(l)\, dl = 1 - \exp\left(-\frac{sL^2}{2k'_+ N}\right)$, and therefore, the probability that all n filaments have lengths less than L is $F_n(L) = \left(1 - \exp\left(-\frac{sL^2}{2k'_+ N}\right)\right)^n$. The probability density function for the longest filament length is given by the derivative of $F_n(L)$,

$$P_n(L) = \frac{d}{dL} F_n(L) = \frac{nsL}{k'_+ N} \left(1 - \exp\left(-\frac{sL^2}{2k'_+ N}\right)\right)^{n-1} \exp\left(-\frac{sL^2}{2k'_+ N}\right). \tag{18}$$

This equation is plotted in Figure S3F.

Autocorrelation Decay Parameter for Bare Filaments, Bundled Structures, and Co-assembly of Structures

In order to calculate the autocorrelation decay, we use Langevin equations associated with different growth mechanisms. For a univariate stochastic process y_t, the autocorrelation at lag k is given by $r_k = \frac{c_k}{c_0}$, where $c_k = \frac{1}{T} \sum_{t=1}^{T-k} (y_t - \bar{y})(y_{t+k} - \bar{y})$ is the lag-k covariance, and c_0 is the sample variance of the time series [67]. According to the Wiener–Khinchin theorem, the autocorrelation function of a stationary process is also the inverse Fourier transform of its power spectral density [65, 66, 68]. Using the techniques outlined in [65, 66, 68], we derive the expressions for the autocorrelation function corresponding to the various growth mechanisms considered here.

bioRxiv preprint doi: https://doi.org/10.1101/2025.05.24.655867; this version posted May 28, 2025. The copyright holder for this preprint (which was not certified by peer review) is the author/funder, who has granted bioRxiv a license to display the preprint in perpetuity. It is made available under aCC-BY 4.0 International license.

Bare Filament

Assembly of a bare filament with constant disassembly in a limited pool: Here, the dynamics of filament length are governed by the following stochastic differential equation:

$$\frac{\mathrm{d}L}{\mathrm{d}t} = k_{+}^{'}(N-L) - k_{-} + \eta, \tag{19}$$

where L denotes the filament length, and η represents a stochastic noise term associated with its dynamics. At steady state, setting the time derivative and the average noise to zero yields the total filament length $L_{\mathrm{ss}} = N - \frac{k_{-}}{k_{+}^{'}}$. To study fluctuations around the steady state, we introduce a small perturbation $L = L_{\mathrm{ss}} + \Delta L$, and substitute this into the dynamic equation for filament length. Since $\frac{\mathrm{d}L_{\mathrm{ss}}}{\mathrm{d}t} = 0$, and using the expression for L_{ss}, we obtain:

$$\frac{\mathrm{d}\Delta L}{\mathrm{d}t} = -k_{+}^{'}\Delta L + \eta. \tag{20}$$

Next, taking the Fourier transform of the dynamical equation yields $i\omega\Delta L(\omega) = -k_{+}^{'}\Delta L(\omega) + \eta(\omega)$, where $\Delta L(\omega)$ and $\eta(\omega)$ are the Fourier transforms of the length fluctuation and the noise, respectively. Rearranging, we obtain $\Delta L(\omega) = \frac{\eta(\omega)}{i\omega + k_{+}^{'}}$, leading to a power spectrum of $|\Delta L(\omega)|^2 = \frac{|\eta(\omega)|^2}{\omega^2 + (k_{+}^{'})^2}$. Assuming delta-correlated noise, $\langle \eta(t)\eta(t')\rangle = 2\eta_0^2\delta(t-t')$, which implies a flat noise spectrum $|\eta(\omega)|^2 = 2\eta_0^2$, we find that the power spectrum of length fluctuations is given by $|\Delta L(\omega)|^2 = \frac{2\eta_0^2}{\omega^2 + (k_{+}^{'})^2}$. By the Wiener-Khinchin theorem, the autocorrelation function is the inverse Fourier transform of the power spectrum, yielding $\mathrm{AC}(t) = e^{-k_{+}^{'}t}$, with an autocorrelation decay parameter $\alpha_{\mathrm{D}} = k_{+}^{'}$. This equation is plotted in Figures 2B–2C and Figure S2A.

The same technique has been employed to determine the autocorrelation decay for the other growth mechanisms as well.

Assembly of a bare filament with severing in a free pool: The filament length dynamics are described by the stochastic differential equation

$$\frac{dL}{dt} = k_{+}^{'}N - \frac{2}{\pi}sL^2 + \eta. \tag{21}$$

Using the methodology outlined above, the autocorrelation decay rate is given by $\alpha_{\mathrm{S}} = \sqrt{\frac{8k_{+}^{'}Ns}{\pi}}$. The corresponding plot of this equation is shown in Figures 2B–2C and Figure S2B.

Assembly of a bare filament with severing in a limited pool: The filament length dynamics are described by the stochastic differential equation

$$\frac{dL}{dt} = k_{+}^{'}(N-L) - \frac{2}{\pi}sL^2 + \eta. \tag{22}$$

Applying the previously described methodology, we obtain the autocorrelation decay rate as $\alpha_{\mathrm{LPS}} = \sqrt{(k_{+}^{'})^2 + \frac{8k_{+}^{'}Ns}{\pi}}$. This equation is plotted in Figure S1F.

bioRxiv preprint doi: https://doi.org/10.1101/2025.05.24.655867; this version posted May 28, 2025. The copyright holder for this preprint (which was not certified by peer review) is the author/funder, who has granted bioRxiv a license to display the preprint in perpetuity. It is made available under aCC-BY 4.0 International license.

Bundled Structure

Assembly of a bundle with severing in a free pool: We analyze the dynamics of a bundle consisting of n filaments, where the length evolution of an individual filament is governed by

$$\frac{\mathrm{d}L_i}{\mathrm{d}t} = \frac{k'_+}{n}N - \frac{2s}{\pi n}L_i^2 + \eta_i, \quad i = 1, \ldots, n, \tag{23}$$

with k'_+N representing the polymerization rate and s denoting the severing rate of the total filament length. Assuming all filaments maintain equal lengths, i.e., $L_i = L_{\text{avg}}$, the dynamics reduce to the following equation for the average filament length:

$$\frac{\mathrm{d}L_{\text{avg}}}{\mathrm{d}t} = \frac{k'_+}{n}N - \frac{2s}{\pi n}L_{\text{avg}}^2 + \eta_{\text{avg}}. \tag{24}$$

Using the same technique described above, we obtain the autocorrelation decay parameter for the average filament length as $\alpha_{\text{S}} = \frac{1}{n}\sqrt{\frac{8k'_+Ns}{\pi}}$. This equation for the average filament length closely agrees with the simulated autocorrelation decay parameter of the bundle length, and the corresponding plots are shown in Figures 4B–4C, S2D, and S4E–S4F.

Assembly of a bundle with severing in a limited pool: Here the length evolution of an individual filament is governed by

$$\frac{\mathrm{d}L_i}{\mathrm{d}t} = \frac{k'_+}{n}\left(N - \sum_{f=1}^{n} L_f\right) - \frac{2s}{\pi n}L_i^2 + \eta_i, \quad i = 1, \ldots, n, \tag{25}$$

with $k'_+(N - L)$ representing the polymerization rate and s denoting the severing rate of the total filament length. Assuming all filaments maintain equal lengths, i.e., $L_i = L_{\text{avg}}$, the dynamics reduce to the following equation for the average filament length:

$$\frac{\mathrm{d}L_{\text{avg}}}{\mathrm{d}t} = \frac{k'_+}{n}(N - nL_{avg}) - \frac{2s}{\pi n}(L_{\text{avg}})^2 + \eta_{\text{avg}}. \tag{26}$$

Using the same technique described above, we obtain the autocorrelation decay parameter for the average filament length as $\alpha_{\text{LPS}} = -k'_+ + \frac{1}{n}\sqrt{(nk'_+)^2 + \frac{8k'_+Ns}{\pi}}$. This equation for the average filament length closely agrees with the simulated autocorrelation decay parameter of the bundle length, and is plotted in Figure S3H.

Co-assembly of Structures

Assembly of multiple filaments regulated by constant disassembly in a limited pool: We consider n filaments, where the total growth rate of all filaments is given by $k'_+(N - L)$, and the disassembly rate is given by k_-. The dynamics of the filament lengths are described by:

$$\frac{\mathrm{d}L_i}{\mathrm{d}t} = \frac{k'_+}{n}\left(N - \sum_{f=1}^{n} L_f\right) - \frac{k_-}{n} + \eta_i, \quad i = 1, \ldots, n. \tag{27}$$

Assuming all filaments in the bundle have equal length, denoted by $L_1 = L_2 = \cdots = L_n = L_{\text{avg}}$, where L_{avg} represents the average length of the n filaments, the total filament length

bioRxiv preprint doi: https://doi.org/10.1101/2025.05.24.655867; this version posted May 28, 2025. The copyright holder for this preprint (which was not certified by peer review) is the author/funder, who has granted bioRxiv a license to display the preprint in perpetuity. It is made available under aCC-BY 4.0 International license.

$L_{\text{tot}} = \sum_{f=1}^{n} L_f$ and the total noise term $\eta_{\text{tot}} = \sum_{f=1}^{n} \eta_f$; the time evolution of the total filament length is then described by:

$$\frac{\mathrm{d}L_{\text{tot}}}{\mathrm{d}t} = k_+^{'}(N - L_{\text{tot}}) - k_- + \eta_{\text{tot}}, \tag{28}$$

By applying the previously outlined method, the autocorrelation decay parameter for the total filament length is found to be $\alpha_{\text{D}} = k_+^{'}$. This equation is plotted in Figure 5E.

Assembly of multiple filaments regulated by severing in a free pool: We consider a system of n filaments, where the length of each filament evolves according to

$$\frac{\mathrm{d}L_i}{\mathrm{d}t} = \frac{k_+'}{n}N - \frac{2s}{\pi n}L_i^2 + \eta_i, \quad i = 1, \ldots, n, \tag{29}$$

with $k_+^{'}N$ representing the total polymerization rate and s the severing rate. Assuming all filaments remain of equal length, $L_i = L_{\text{avg}}$, the dynamics of the total filament length $L_{\text{tot}} = \sum_{i=1}^{n} L_i$ simplify to

$$\frac{\mathrm{d}L_{\text{tot}}}{\mathrm{d}t} = k_+^{'}N - \frac{2s}{\pi n^2}L_{\text{tot}}^2 + \eta_{\text{tot}}, \tag{30}$$

where $\eta_{\text{tot}} = \sum_{i=1}^{n} \eta_i$ represents the cumulative noise from all filaments. Applying the same Fourier-based analysis as described earlier, we obtain the autocorrelation decay rate for the total filament length as $\alpha_{\text{S}} = \frac{1}{n}\sqrt{\frac{8k_+^{'}Ns}{\pi}}$. This equation is plotted in Figure 5E.

To analyze the relative dynamics between filaments, we consider the length difference between any two filaments i and j, denoted by $L_{\text{diff}} = L_{ij} = L_i - L_j$. By subtracting the corresponding equations for L_i and L_j, the dynamics of the filament length difference are given by:

$$\frac{dL_{\text{diff}}}{dt} = -\frac{2s}{\pi n}\left[L_i^2 - L_j^2\right] + \eta_{\text{diff}}. \tag{31}$$

Applying the same method described above, we derive the autocorrelation decay parameter for the filament length difference, which is the same as the expression derived for the total filament length: $\alpha_{\text{S}} = \frac{1}{n}\sqrt{\frac{8k_+^{'}Ns}{\pi}}$. This equation is plotted in Figure 5E.

Assembly of multiple filaments regulated by severing in a limited pool: Here, we analyze a system consisting of n filaments, where the growth of each filament is regulated by both a shared monomer pool and severing. The evolution equation for the length of the ith filament is given by:

$$\frac{\mathrm{d}L_i}{\mathrm{d}t} = \frac{k_+^{'}}{n}\left(N - \sum_{f=1}^{n} L_f\right) - \frac{2s}{\pi n}L_i^2 + \eta_i, \quad i = 1, \ldots, n, \tag{32}$$

where $k_+^{'}$ is the polymerization rate per filament and s denotes the severing rate. Assuming uniform filament lengths, i.e., $L_i = L_{\text{avg}}$ for all i, the total filament length $L_{\text{tot}} = \sum_{i=1}^{n} L_i$ follows the reduced form:

$$\frac{\mathrm{d}L_{\text{tot}}}{\mathrm{d}t} = k_+^{'}(N - L_{\text{tot}}) - \frac{2s}{\pi n^2}L_{\text{tot}}^2 + \eta_{\text{tot}}, \tag{33}$$

with $\eta_{\text{tot}} = \sum_{i=1}^{n} \eta_i$ capturing the collective noise across all filaments. By applying the Fourier-based approach used in previous sections, the autocorrelation decay rate for the total filament length is found to be $\alpha_{\text{LPS}} = \frac{1}{n}\sqrt{(nk_+^{'})^2 + \frac{8k_+^{'}Ns}{\pi}}$.

To further understand the internal dynamics of the system, we consider the difference in

bioRxiv preprint doi: https://doi.org/10.1101/2025.05.24.655867; this version posted May 28, 2025. The copyright holder for this preprint (which was not certified by peer review) is the author/funder, who has granted bioRxiv a license to display the preprint in perpetuity. It is made available under aCC-BY 4.0 International license.

length between two filaments, defined as $L_{\text{diff}} = L_i - L_j$. Subtracting their individual length evolution equations yields the following expression:

$$\frac{\mathrm{d}L_{\text{diff}}}{\mathrm{d}t} = -\frac{2s}{\pi n}\left(L_i^2 - L_j^2\right) + \eta_{\text{diff}}. \tag{34}$$

Applying the same analytical technique, we derive the autocorrelation decay rate for this length difference as $\alpha_{\text{LPS}} = -k'_+ + \frac{1}{n}\sqrt{(nk'_+)^2 + \frac{8k'_+Ns}{\pi}}$.

Assembly of multiple bundles regulated by severing: We consider b bundles with n filaments each, resulting in a total of nb filaments. Using the Fourier-based technique outlined above, the autocorrelation decay parameter for a free pool is given by $\alpha_{\text{S}} = \frac{1}{nb}\sqrt{\frac{8k'_+Ns}{\pi}}$. This equation is plotted in Figure 5F. For a limited pool, the decay parameter becomes $\alpha_{\text{LPS}} = \frac{1}{nb}\sqrt{(nbk'_+)^2 + \frac{8k'_+Ns}{\pi}}$, which is shown in Figure S5B.

Simulation Protocol

We employed Gillespie stochastic simulations to model the growth of bare filaments and bundles. A bundle is defined as a collection of linear, parallel filaments, with its length determined by the maximum length among them. For both bare filaments and bundles, simulations begin with all filaments initialized at zero length. At each simulation step, a possible transition—corresponding to a change in filament length—is randomly selected with a probability proportional to its associated rate. The time interval between transitions is drawn from an exponential distribution, whose rate parameter equals the sum of all possible transition rates from the current state. This process is repeated until the system reaches a steady-state length distribution. To ensure statistical reliability, we generate multiple independent stochastic trajectories and compute the steady-state distributions of filament and bundle lengths by averaging over these realizations.

To analyze fluctuations in the steady-state time series of filament and bundle lengths, we computed the autocorrelation using MATLAB's `autocorr` function.

bioRxiv preprint doi: https://doi.org/10.1101/2025.05.24.655867; this version posted May 28, 2025. The copyright holder for this preprint (which was not certified by peer review) is the author/funder, who has granted bioRxiv a license to display the preprint in perpetuity. It is made available under aCC-BY 4.0 International license.

Supplemental Results

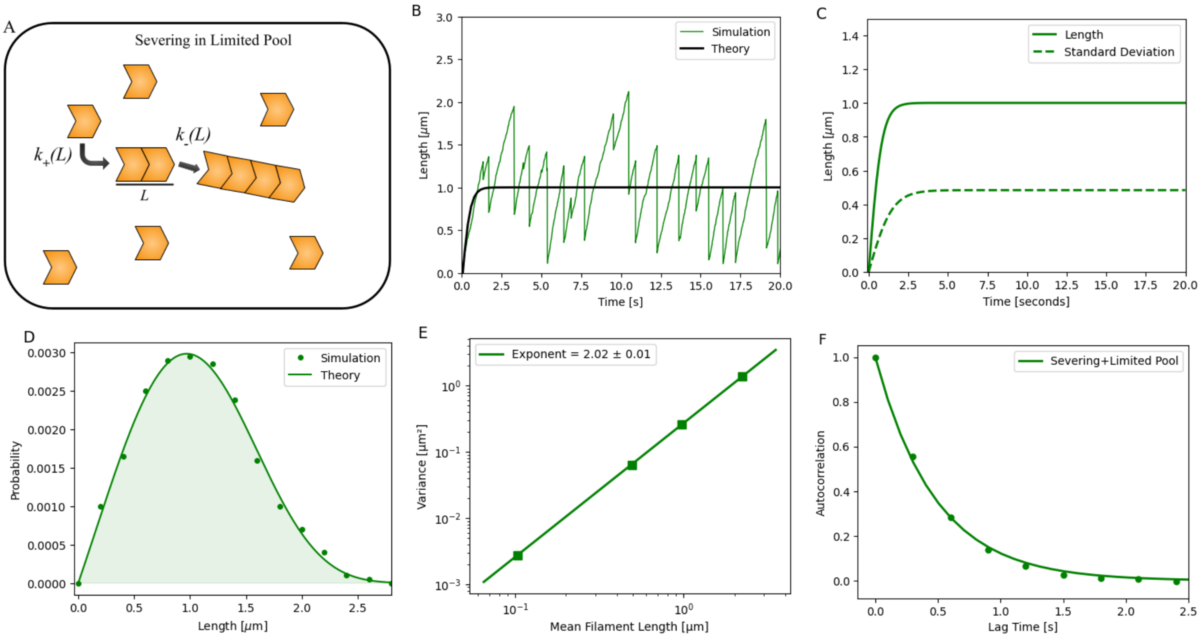

Figure S1. Growth dynamics of a bare filament under severing in a limited pool.
(A) Schematic illustrating the growth of a bare filament.
(B) Stochastic simulations of filament growth trajectories. Simulation results are overlaid with analytical predictions (black), as described in the Supplementary Material.
(C) Mean length (solid lines) and standard deviation (dashed lines) of bare filaments, calculated from simulations, plotted as a function of time.
(D) Probability distribution of steady-state filament lengths. Dots represent simulation data; solid lines indicate analytical predictions (see Supplementary Material).
(E) Variance of filament length distributions plotted against the corresponding mean filament lengths (log-log scale).
(F) Autocorrelation of steady-state filament length over time. Dots represent simulation results; lines show analytical predictions (see Supplementary Material for detailed derivations).
Parameters: $N = 1000$ monomers (each $4\,\mathrm{nm}$ in size), $k'_+ = 0.3\ \mathrm{s}^{-1}$, and $s = 0.00565\ \mathrm{monomers}^{-1}\ \mathrm{s}^{-1}$. For panel (E), different values of k'_+ were selected to obtain distinct mean filament lengths, with $N = 10000$: $k'_+ = (0.005, 0.125, 0.5, 2.75)\ \mathrm{s}^{-1}$ and $s = 0.125\ \mathrm{monomers}^{-1}\ \mathrm{s}^{-1}$.

bioRxiv preprint doi: https://doi.org/10.1101/2025.05.24.655867; this version posted May 28, 2025. The copyright holder for this preprint (which was not certified by peer review) is the author/funder, who has granted bioRxiv a license to display the preprint in perpetuity. It is made available under aCC-BY 4.0 International license.

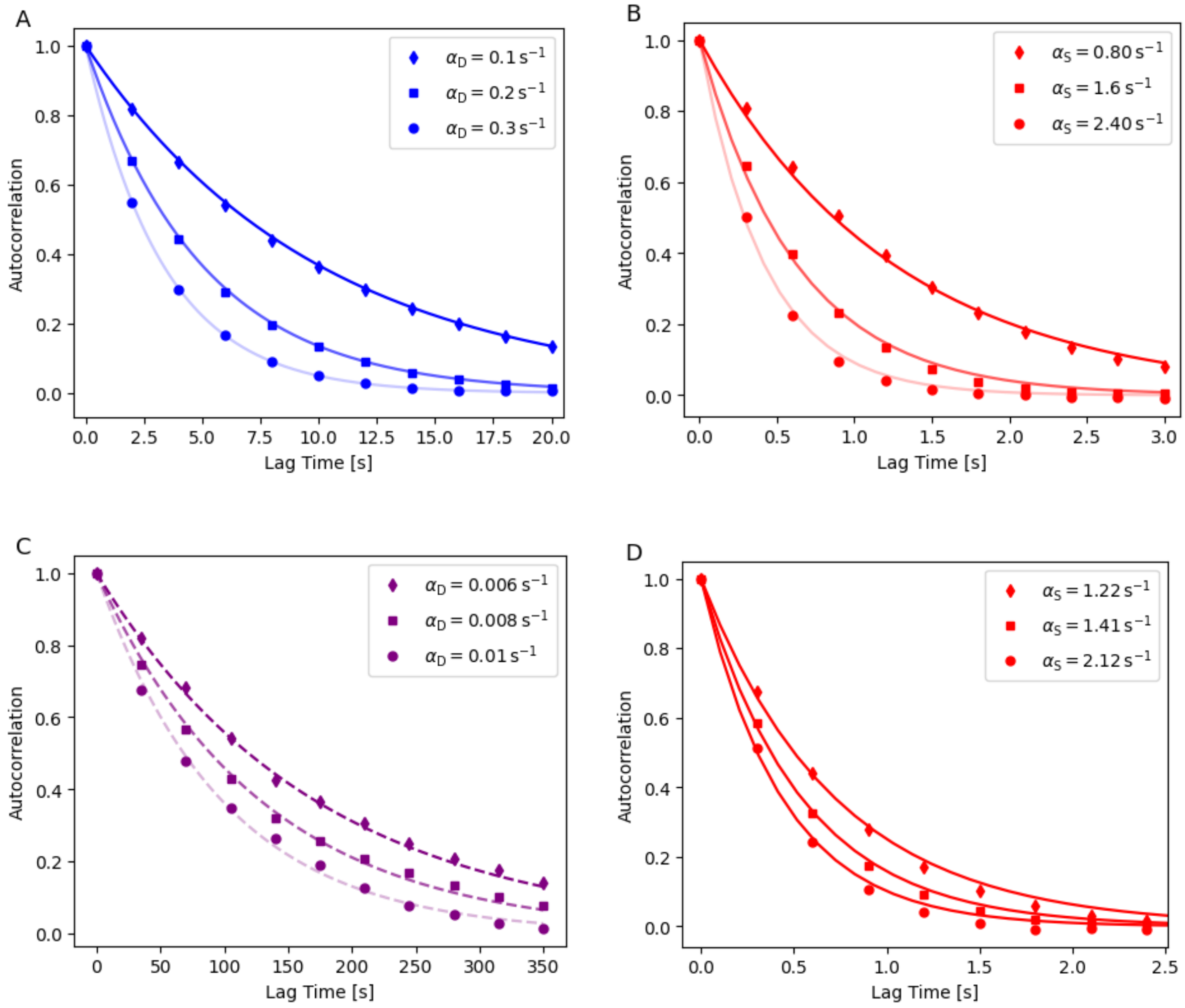

Figure S2. Autocorrelation analysis of steady-state length fluctuations in single filaments and bundles under various assembly-disassembly mechanisms.
(A–B) Filaments: Autocorrelation of filament length over time for different values of α, with $N = 1000$ and mean filament length maintained near $1\ \mu$m using (A) constant disassembly in a limited pool and (B) severing in a free pool.
(C–D) Bundles: Corresponding autocorrelation plots for bundle length with $N = 100000$, again maintaining $\sim 1\ \mu$m mean filament length, under (C) constant disassembly in a free pool and (D) severing in a free pool. Dots represent simulation data; solid lines are analytical predictions, and the dashed line is the fitted result (see Supplementary Material for derivation details).

bioRxiv preprint doi: https://doi.org/10.1101/2025.05.24.655867; this version posted May 28, 2025. The copyright holder for this preprint (which was not certified by peer review) is the author/funder, who has granted bioRxiv a license to display the preprint in perpetuity. It is made available under aCC-BY 4.0 International license.

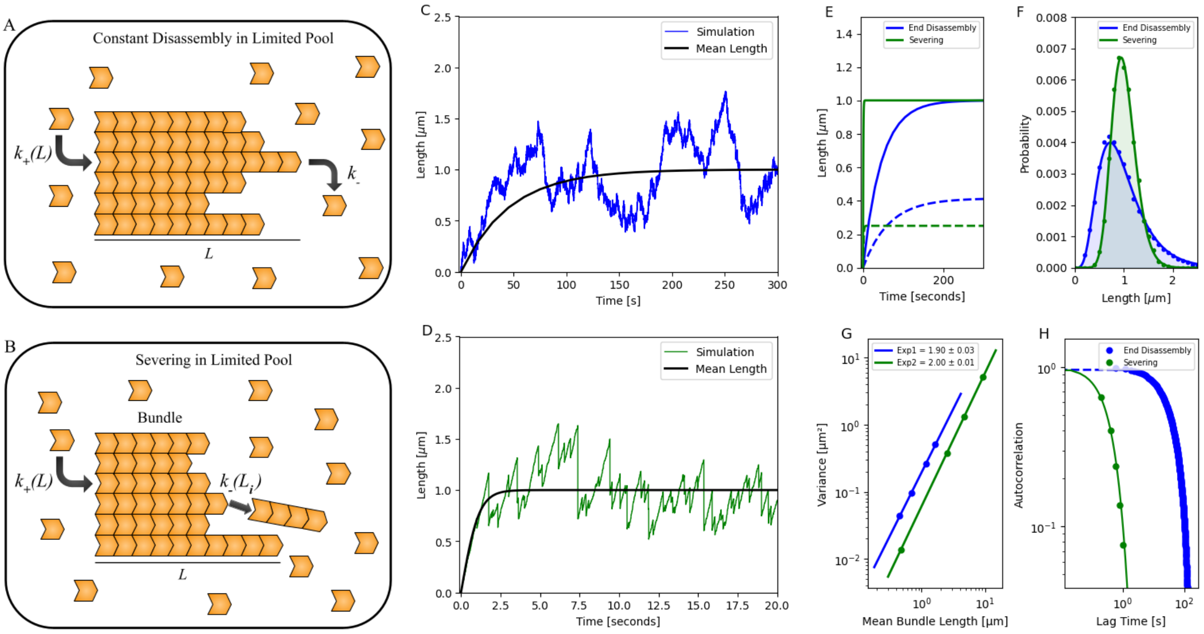

Figure S3. Growth dynamics of a bundle in a limited pool with (1) constant disassembly and (2) severing.
(A, B) Schematics showing the growth of a bundle in a limited pool with: (1) constant disassembly and (2) severing.
(C, D) Stochastic simulations of bundle growth regulated limited pool with (1) constant disassembly and (2) severing. After an initial growth phase, bundle length reaches a steady state. Simulation results are compared with fitted results (black), guided by analytical calculations described in the Supplementary Material.
(E) Stochastic simulations of the mean bundle length (solid line) and standard deviation (dotted line) over time for both mechanisms.
(F) Probability distribution of steady-state bundle lengths for the two mechanisms. Dots indicate simulation data, and solid lines represent analytical results (see Supplementary Material).
(G) Variance of bundle length distributions plotted against the mean bundle lengths. Both mechanisms are shown on a log-log scale.
(H) Autocorrelation of steady-state bundle length over time for the two mechanisms, shown on a log-log scale. Dots represent simulation results, solid lines indicate analytical predictions, and dashed lines denote fitted data (see Supplementary Material).
Parameters for panels (C–F): $N = 100{,}000$ monomers (each $4\,\mathrm{nm}$ in size), with $k'_+ = 0.01\ \mathrm{s}^{-1}$. For the limited pool with end disassembly, $k_- = 1002.8\ \mathrm{s}^{-1}$; for the limited pool with severing, $s = 0.074\ \mathrm{monomers}^{-1}\ \mathrm{s}^{-1}$. In panel (G), simulations were performed with $N = 200{,}000$ monomers and a constant assembly rate $k'_+ = 0.06\ \mathrm{s}^{-1}$ across all cases. For the limited pool with end disassembly, disassembly rates were $k_- = \{12005,\ 12050,\ 12150,\ 12250\}\ \mathrm{s}^{-1}$; for the limited pool with severing, severing rates were $s = \{0.01,\ 0.04,\ 0.14,\ 4\}\ \mathrm{monomers}^{-1}\ \mathrm{s}^{-1}$.

bioRxiv preprint doi: https://doi.org/10.1101/2025.05.24.655867; this version posted May 28, 2025. The copyright holder for this preprint (which was not certified by peer review) is the author/funder, who has granted bioRxiv a license to display the preprint in perpetuity. It is made available under aCC-BY 4.0 International license.

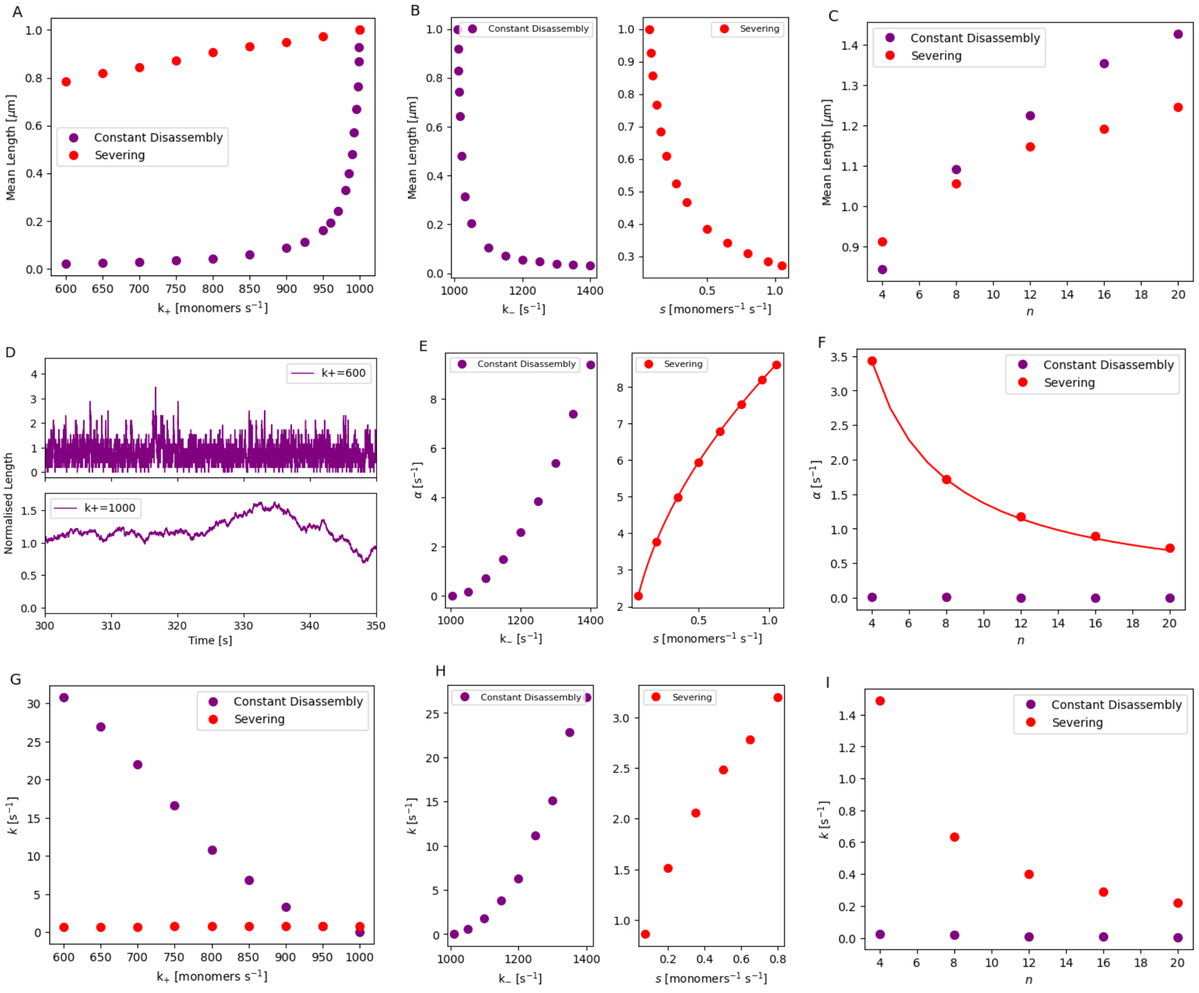

Figure S4. Parameter dependence of length, autocorrelation decay, and relaxation rate for bundles in a free monomer pool regulated by (1) constant disassembly and (2) severing.
(A–C) Steady-state bundle length for the two mechanisms plotted as a function of k_+, k_-, and n, respectively, with other parameters held constant.
(D) Simulated time trajectory of the steady-state bundle length regulated by simple assembly for $k_+ = 600$ and 1000, respectively, with other parameters held constant.
(E–F) Autocorrelation decay parameter of the steady-state bundle length for the two mechanisms plotted as a function of k_-, and n, respectively. Dots represent simulation results, and lines indicate analytical predictions (see Supplementary Material for detailed derivations).
(G–I) Relaxation rate toward the steady-state bundle length for the two mechanisms plotted as a function of k_+, k_-, and n, respectively, with other parameters held constant.
Parameters: $N = 100{,}000$ monomers (each $4\,\mathrm{nm}$ in size), $k'_+ = 0.01\ \mathrm{s}^{-1}$; for simple assembly, $k_- = 1010\ \mathrm{s}^{-1}$; for the free pool with severing, $s = 0.074\ \mathrm{monomers}^{-1}\ \mathrm{s}^{-1}$.

bioRxiv preprint doi: https://doi.org/10.1101/2025.05.24.655867; this version posted May 28, 2025. The copyright holder for this preprint (which was not certified by peer review) is the author/funder, who has granted bioRxiv a license to display the preprint in perpetuity. It is made available under aCC-BY 4.0 International license.

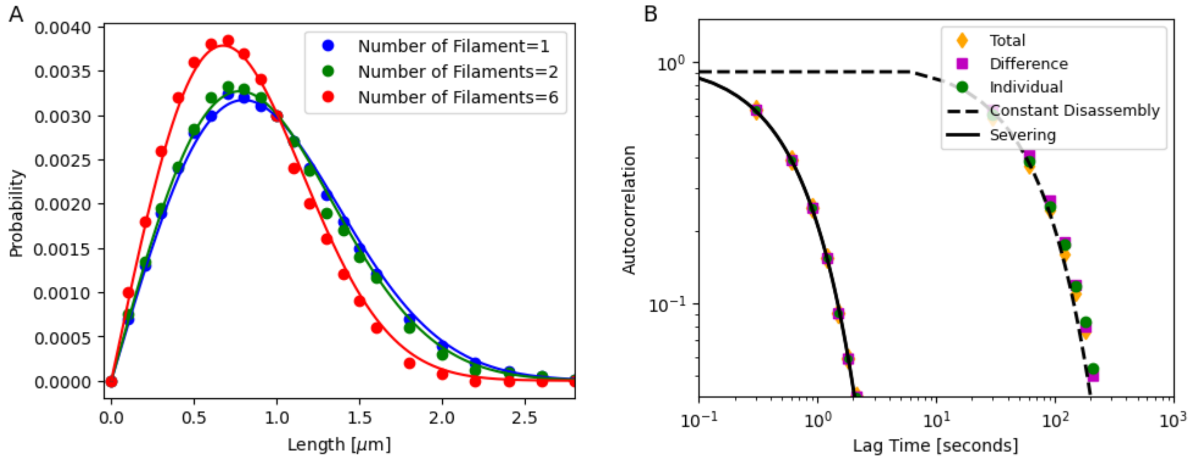

Figure S5. Length distribution and autocorrelation analysis of multiple filaments and bundles under various assembly-disassembly mechanisms.
(A) Steady-state length distributions for $n = 1, 2, 6$ filaments regulated by severing in limited pool. Dots represent simulation results, while lines indicate analytical predictions (see Supplementary Material). Parameters: $N = 3000$ (monomers, each 4 nm in size) , $k'_+ = 0.05\,\mathrm{s}^{-1}$, and $s = 0.00375$ monomers^{-1} s^{-1}.
(B) Autocorrelation functions for two co-assembling bundles in limited pool under constant disassembly (dashed lines) and severing (solid lines) mechanisms, showing individual filaments, their sum, and their difference. The plots are shown on a log-log scale, with dots representing simulation results, black solid and dashed lines indicating analytical predictions and fitted data, respectively (see Supplementary Material).
Parameters: each bundle contains 4 filaments, with $N = 100{,}000$, $k'_+ = 0.01\,\mathrm{s}^{-1}$, $k_- = 1008.2\,\mathrm{s}^{-1}$ (constant disassembly), and $s = 0.062$ monomer^{-1} s^{-1} (severing).

bioRxiv preprint doi: https://doi.org/10.1101/2025.05.24.655867; this version posted May 28, 2025. The copyright holder for this preprint (which was not certified by peer review) is the author/funder, who has granted bioRxiv a license to display the preprint in perpetuity. It is made available under aCC-BY 4.0 International license.

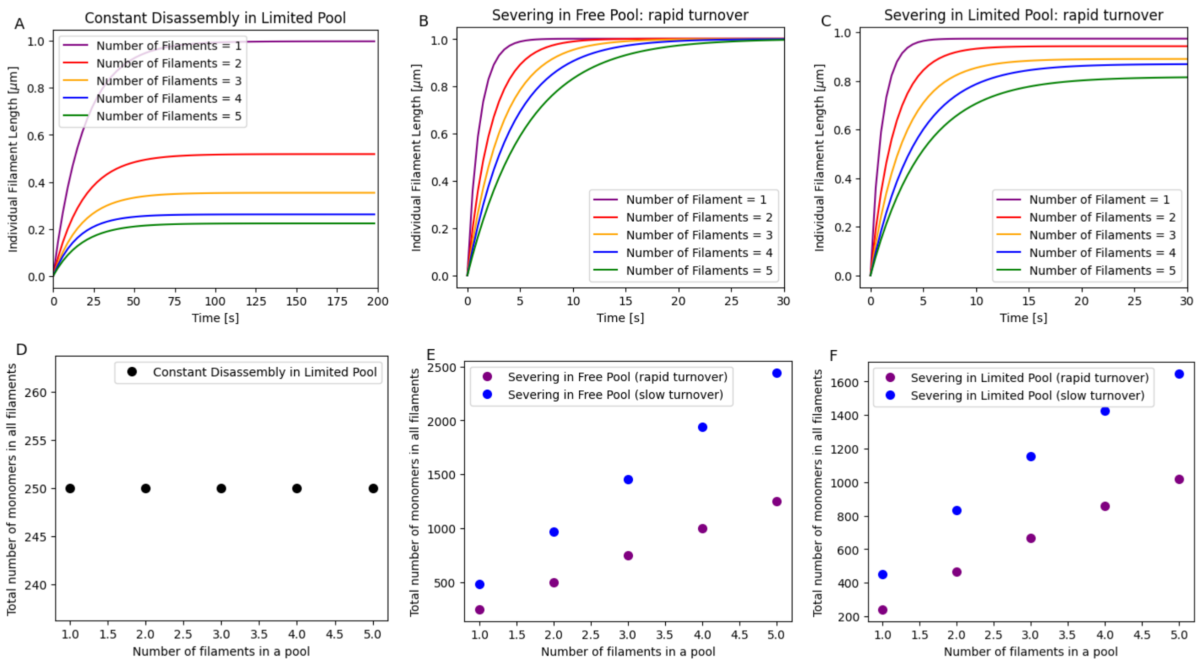

Figure S6. Assembly dynamics of multiple filaments under different conditions: (1) constant disassembly in a limited pool, (2) severing in a free pool, and (3) severing in a limited pool.
(A–C) Stochastic simulations of the mean length of multiple filaments over time for each mechanism.
(D–F) Total number of monomers incorporated into all filaments for each mechanism.
Parameters: $N = 3000$ monomers (each $4\,\text{nm}$ in size), with $k'_+ = 0.05\ \text{s}^{-1}$ for all mechanisms. For the constant disassembly in a limited pool, $k_- = 137.5\ \text{s}^{-1}$; for the severing in a free pool and the severing in a limited pool, severing rates were $s = 0.00375\ \text{monomers}^{-1}\ \text{s}^{-1}$ (rapid turnover) and $s = 0.001\ \text{monomers}^{-1}\ \text{s}^{-1}$ (slow turnover).

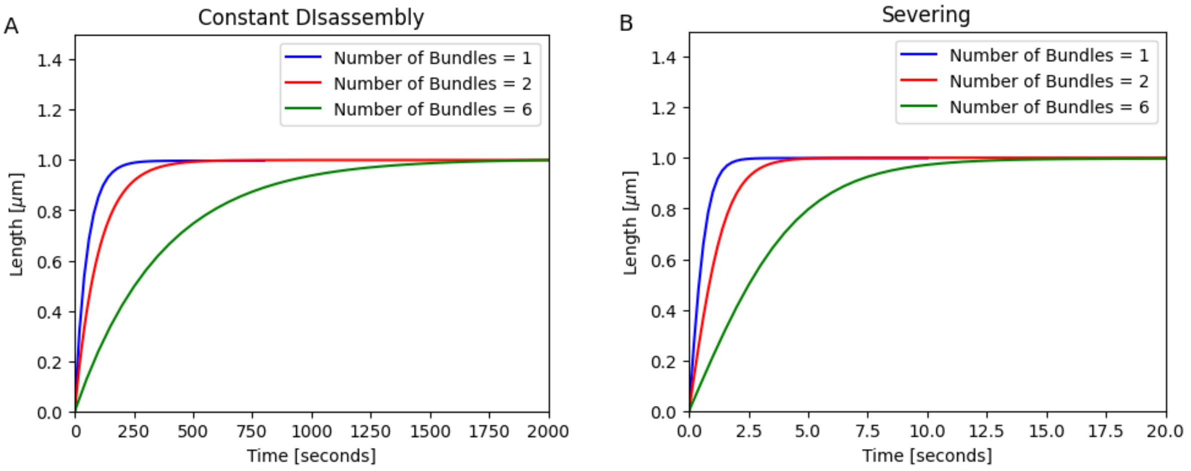

Figure S7. Growth dynamics of multiple bundles in a free pool with (1) constant disassembly and (2) severing mechanisms.
(A–B) Stochastic simulations of the mean bundle length over time for both mechanisms.
Parameters: each bundle contains 4 filaments, with $N = 100{,}000$ monomers (each $4\,\text{nm}$ in size), and $k'_+ = 0.01\ \text{s}^{-1}$. For constant disassembly, $k_- = 1008.2\ \text{s}^{-1}$; for the severing, $s = 0.062\ \text{monomers}^{-1}\ \text{s}^{-1}$.
